# Evaluating Ketamine Dosing Practices During Emergency Department Intubation Using Shock Index Stratification

**DOI:** 10.7759/cureus.109356

**Published:** 2026-05-21

**Authors:** Katelyn Gowlett, Robin Clouston, Paul Atkinson, Jay Mekwan, Pamela McDougall, Kavish Chandra

**Affiliations:** 1 Faculty of Medicine, Dalhousie Medicine New Brunswick, Saint John, CAN; 2 Department of Emergency Medicine, Dalhousie University, Saint John, CAN; 3 Department of Emergency Medicine, Saint John Regional Hospital, Saint John, CAN

**Keywords:** emergency medicine research, ketamine dosing, medication administration, rapid sequence induction, shock index, endotracheal intubation

## Abstract

Background

The accurate dosing of ketamine during rapid sequence intubation in emergency settings is critical, especially in patients with varying hemodynamic states. This study describes ketamine dosing practices during rapid sequence intubation in a tertiary emergency department, focusing on concordance with predefined dosing ranges based on shock index. The primary goal is to analyze registry data to assess concordance with recommended weight-based dosing ranges, considering shock index and shock dosing.

Methods

We analyzed intubation data from the Emergency Medicine - Airway Registry (EM-AWARE) from 2015 to 2021. We included adult patients aged 18 years and above who underwent intubation with ketamine as the sole sedative agent, and stratified patients into two groups: those with a normal shock index below 0.9 and those with an abnormal shock index at or above 0.9. Data were analyzed to determine the proportion of patients who received ketamine doses within, below, or above predefined dosing ranges, and results were compared across the two groups.

Results

Forty-six patients met the inclusion criteria. There were 29 patients in the normal shock index group, and among these patients, 72% (n = 21) received a dose within the predefined range, and 28% (n = 8) received doses below the range. There were 17 patients in the abnormal shock index group, with 76% (n = 13) receiving a reduced dose within the predefined range and 24% (n = 4) receiving doses outside the range, primarily above the recommended shock dosing range. Overall rates of dosing outside predefined ranges were similar between groups, ranging from 24% to 28%.

Conclusion

In this single-centre registry analysis, approximately one-quarter of emergency department patients received ketamine doses outside predefined ranges during intubation, regardless of shock index. These findings highlight persistent variability in ketamine dosing practices during emergency airway management.

## Introduction

Endotracheal intubation is a high-acuity, variable-frequency procedure in Emergency Departments. It is a mainstay in critically ill patients who require advanced airway management. It involves multiple time-critical steps, each vulnerable to cognitive overload and error. Emergency physicians are tasked with making rapid and precise decisions regarding the care of these patients. According to specific practice guidelines, successful intubation can encompass approximately 31 distinct steps [1], each of which is crucial for achieving the desired patient outcome.

The cognitive demands placed on the emergency medicine team during intubation are immense, as they must simultaneously manage the patient’s airway, monitor vital signs, and make rapid pharmacological calculations. This increased cognitive load can impair decision-making processes and lead to medication errors [2,3]. Errors in medication administration processes, whether they result in underdosing or overdosing, can have significant repercussions [4]. Such errors can lead to adverse patient outcomes, including increased morbidity and mortality, especially in patients who are hemodynamically unstable [5,6]. Ensuring the precise dosing of medications is paramount to minimizing these risks and promoting patient safety.

Ketamine is a commonly used induction agent for rapid sequence intubation due to its dissociative anesthetic properties, rapid onset, and perceived hemodynamic stability [7]. In patients who are hemodynamically unstable, the practice of administering reduced or “shock-dose” ketamine has been adopted to mitigate peri-intubation hypotension and cardiovascular collapse [8]. Observational studies comparing ketamine to alternative induction agents have demonstrated clinically significant rates of peri-intubation hypotension following ketamine administration [9,10]. These findings suggest that critically ill patients may be vulnerable to ketamine’s dose-dependent hemodynamic effects. There is an existing gap in the literature of the intersection of these two topics, specifically clinical analysis and application of ketamine dosing variability within the context of shock index.

This study aims to analyze emergency department registry data to assess ketamine dosing during endotracheal intubation at the Saint John Regional Hospital, specifically examining concordance with predefined weight-based dosing ranges according to patient shock index.

This study was previously presented as a poster at the New Brunswick Health Research Symposium on November 14, 2024.

## Materials and methods

Study design 

We conducted a retrospective observational study using data from the Emergency Medicine - Airway Registry (EM-AWARE).

Study setting and population 

The EM-AWARE is a retrospective, observational, and self-reported collection of data on emergency department airway interventions and associated outcomes. This database includes records from 2015 to 2021, compiled at the Saint John Regional Hospital Emergency Department, a Level 1 trauma centre within a tertiary hospital, with an annual emergency department census of approximately 60,000 patients. Data are entered into the database by the lead physician following emergency department intubation and include demographic, pre-intubation, peri-intubation, and post-intubation variables. Missing data was secondarily added where available by trained abstractors. For study inclusion, only records with all complete variables of interest were included in the final analysis.

We included all adult patients aged 18 years and older who required airway management and intubation using ketamine as a single sedative agent for induction, with or without concomitant paralytic use. We divided patients into two groups: those with a normal shock index below 0.9 and those with an abnormal shock index at or above 0.9.

We excluded pediatric patients, those with cardiac arrest preceding intubation, patients who received sedative medications other than ketamine or additional sedatives during induction, and those with incomplete data. Recorded patient weight reflected documented or estimated values as captured in the registry, consistent with routine emergency department practice. Methods used for weight measurements included estimations, patient-reported weights, and measured weights; however, this was not explicitly stratified within the dataset.

Outcomes 

The primary outcome of interest was concordance of administered ketamine doses with predefined weight-based dosing ranges stratified by shock index.

Shock index was used as a surrogate measure of hemodynamic instability and is correlated with increased mortality and adverse outcomes [11]. Shock index was calculated as heart rate divided by systolic blood pressure [12]. Normal shock index values typically range from 0.5 to 0.7, with higher values indicating increasing hemodynamic compromise. In this study, an abnormal shock index was defined as at or above 0.9, a threshold supported in the literature and associated with increased mortality [11,13].

For patients with a normal shock index, appropriate ketamine induction dosing was defined as 1 to 2 mg/kg intravenously administered as a single dose during intubation [14-16]. For patients with an abnormal shock index, appropriate reduced or “shock dosing” was defined as 0.5 to 1 mg/kg intravenously [7,17,18].

Data analysis 

Eligible cases were stratified into normal and abnormal shock index groups. Each case was reviewed to determine whether the administered ketamine dose fell within, below, or above the predefined dosing range for the corresponding shock index category. Descriptive analyses were performed to calculate the proportion of patients in each dosing category; additionally, a Chi-square test was performed through GraphPad (Dotmatics, Boston, MA, USA) to further support the descriptive analysis through inferential statistics.

Ethics

This study was approved by the Horizon Health Research Ethics Board (File No: 101266).

## Results

A total of 46 patients were included in the analysis. Forty-one percent were female patients, and the mean patient weight was 82.7 kg (Table 1). The indication for intubation was medical in 31 cases (67%) and surgical or trauma-related in 14 cases (30%). The most common reason for intubation was reduced Glasgow Coma Scale score in 29 cases (63%), followed by respiratory failure in 14 cases (30%) and worsening clinical course in two cases (4%).

**Table 1:** Sample demographics and data

Variable	Total reported (%) (n = 46)	Total reported (n)	Not reported (n)
Female	41%	19	0
Weight (kg), mean	-	82.71	0
Indication	-	-	1
Medical	67%	31	-
Surgical/trauma	30%	14	-
Reason for intubation	-	-	1
Reduced GCS	63%	29	-
Respiratory failure	30%	14	-
Worsening clinical course	4%	2	-
Impression difficult airway	-	-	0
Yes	37%	17	-
No	63%	29	-
Intubation urgency grading	-	-	1
Emergent	33%	15	-
Urgent	61%	28	-
Semi-urgent	4%	2	-
Device	-	-	0
Laryngoscope	7%	3	-
Video laryngoscope	93%	43	-
Paralytic agent	-	-	0
Rocuronium	96%	44	-
Succinylcholine	2%	1	-
No paralytic given	2%	1	-
Abnormal shock index	37%	17	-
Normal shock index	63%	29	-
Abnormal shock index dosing	(n = 17)	-	-
Within dosing range	76%	13	-
Below dosing range	6%	1	-
Above dosing range	18%	3	-
Normal shock index dosing	(n = 29)	-	-
Within dosing range	72%	21	-
Below dosing range	28%	8	-
Above dosing range	0%	0	-

Twenty-nine patients (63%) had a normal shock index, while 17 patients (37%) had an abnormal shock index. In the normal shock index group, 21 patients (72%) received ketamine doses within the predefined range, while eight patients (28%) received doses below the range. In the abnormal shock index group, 13 patients (76%) received reduced ketamine doses within the predefined range, while four patients (24%) received doses outside the range. Overall, approximately one-quarter of patients in each group received ketamine doses outside predefined ranges. The two-tailed P value, following the Chi-square test, was 0.76, demonstrating no statistical significance between the normal shock index group and the abnormal shock index group.

## Discussion

Interpretation 

In this study, we observed similar rates of ketamine dosing outside predefined ranges in patients with normal and abnormal shock indices. The comparable proportions of dosing variability across shock index groups suggest that physiologic risk stratification alone may be insufficient to consistently guide induction dosing under emergency conditions. These findings likely reflect the complex cognitive and systems-level challenges inherent to emergency airway management.

Previous studies 

Ketamine Dosages 

Prior observational studies have raised concerns regarding ketamine’s hemodynamic effects during rapid sequence intubation, particularly in critically ill patients. Large registry analyses have demonstrated associations between ketamine use and peri-intubation hypotension compared with etomidate [9,10]. Unlike prior studies focused on post-intubation hemodynamic outcomes, the present analysis examines real-world dosing practices relative to physiologic risk stratification.

Error Rates 

Medication error rates in the emergency department vary widely in the literature, ranging from 4% to nearly 60% depending on methodology and definitions [19,20]. Despite this variability, there is a broad consensus that medication errors represent an important patient safety concern. Even isolated errors can result in adverse outcomes [21], supporting the principle that dosing variability during high-risk procedures warrants focused attention.

Strengths and limitations 

A key strength of this study is the use of a comprehensive airway registry capturing detailed peri-intubation variables across several years of emergency department practice. However, several limitations must be acknowledged. This was a single-centre, retrospective registry study with a small sample size, limiting statistical power, generalizability, and the ability to detect significant differences between the studied groups. For this reason, we recommend further studies with a larger sample size to increase the strength and generalizability of the findings. The study was not designed to evaluate clinical outcomes such as hypotension or mortality and lacks rigorous inferential statistical analysis. Reliance on self-reported registry data introduces potential reporting bias and selection bias. Measurement bias is acknowledged, as weight estimation/measurement methods were not documented in our data set. Through mixed methods of weight estimation and measurement, these values had the potential to be inaccurately measured. Additionally, heaping bias may have been present within the data, although it is challenging to validate, as the data set does not concurrently examine estimated weights alongside actual weights. This highlights the challenges with weight accuracy in the emergency department, where estimated weights are frequently used but not consistently validated [22].

Clinical implications 

The observed dosing variability highlights the potential risk of medication error during emergency department intubation. System-level interventions such as standardized dosing aids, improved access to accurate weight estimation, education [23], and cognitive offloading tools may help reduce variability [24]. In high-acuity environments, variability in experience, task load, and available information may all contribute to dosing challenges. Practically, the elimination of estimated weights is recommended as a safety practice, although this may be challenging without an alternative method of measurement. Tools such as weigh beds and weight-based dosing calculators are preferred methods of measurement for accuracy and cognitive offloading [25].

Research implications 

Future research should focus on identifying modifiable contributors to dosing variability during emergency airway management. Larger multi-centre studies would improve statistical power and generalizability. Evaluation of standardized dosing prompts or automated weight-based calculation tools may further inform strategies to reduce medication-related harm.

## Conclusions

This study provides a descriptive examination of ketamine dosing during emergency department intubation, demonstrating that approximately one-quarter of patients received doses outside predefined ranges regardless of shock index. These findings underscore persistent dosing variability and support the need for system-level approaches to improve medication safety. Given the limitations of the study, we suggest larger, multi-centre studies to improve the generalizability and strength of the findings.
